# A prospective multicenter phase II study evaluating multimodality treatment of patients with peritoneal carcinomatosis arising from appendiceal and colorectal cancer: the COMBATAC trial

**DOI:** 10.1186/1471-2407-13-67

**Published:** 2013-02-07

**Authors:** Gabriel Glockzin, Justine Rochon, Dirk Arnold, Sven A Lang, Frank Klebl, Florian Zeman, Michael Koller, Hans J Schlitt, Pompiliu Piso

**Affiliations:** 1 Department of Surgery, University Medical Center Regensburg, Regensburg 93042, Germany; 2 Institute of Medical Biometry and Informatics, University of Heidelberg, Heidelberg, Germany; 3 Department of Medical Oncology, Tumor Biology Clinic, Albert Ludwigs University, Freiburg, Germany; 4 Department of Internal Medicine, University Medical Center Regensburg, Regensburg, Germany; 5 Center for Clinical Studies, University Medical Center Regensburg, Regensburg, Germany; 6 Department of Surgery, St. John of God Hospital Regensburg, Regensburg, Germany

**Keywords:** Cytoreductive surgery, HIPEC, Perioperative chemotherapy, Cetuximab, Colorectal cancer, Peritoneal carcinomatosis

## Abstract

**Background:**

Peritoneal carcinomatosis is regarded as a common sign of advanced tumor stage, tumor progression or local recurrence of appendiceal and colorectal cancer and is generally associated with poor prognosis. Although survival of patients with advanced stage CRC has markedly improved over the last 20 years with systemic treatment, comprising combination chemotherapy +/− monoclonal antibodies, the oncological outcome—especially of the subgroup of patients with peritoneal metastases—is still unsatisfactory. In addition to systemic therapy, cytoreductive surgery (CRS) and hyperthermic intraperitoneal chemotherapy (HIPEC) are specific treatment options for a selected group of these patients and may provide an additional therapeutic benefit in the framework of an interdisciplinary treatment concept.

**Methods/design:**

The COMBATAC trial is a prospective, multicenter, open-label, single-arm, single-stage phase II trial investigating perioperative systemic polychemotherapy including cetuximab in combination with CRS and HIPEC patients with histologically proven wild-type KRAS colorectal or appendiceal adenocarcinoma and synchronous or metachronous peritoneal carcinomatosis. The planned total number of patients to be recruited is 60. The primary endpoint is progression-free survival (PFS). Secondary endpoints include overall survival (OS), perioperative morbidity and treatment-associated toxicity, feasibility of the combined treatment regimen, quality of life (QoL) and histopathological regression after preoperative chemotherapy.

**Discussion:**

The COMBATAC trial is designed to evaluate the feasibility and efficacy of the combined multidisciplinary treatment regimen consisting of perioperative systemic combination chemotherapy plus cetuximab and CRS plus bidirectional HIPEC with intraperitoneal oxaliplatin.

**Trial registration:**

ClinicalTrials.gov Identifier: NCT01540344, EudraCT number: 2009-014040-11

## Background

### Disease under study

Colorectal cancer (CRC) is the third most commonly diagnosed cancer in males and the second in female worldwide and overall the fourth leading cause of cancer-related death. Whereas the mortality associated with CRC slightly decreased over the past 20 years the incidence is still increasing in most countries [1,2]. More than 10% of patients with CRC already show peritoneal carcinomatosis at the time of initial diagnosis [3]. In about 25% of the cases there is no evidence of further distant metastasis [4]. Moreover, up to 25% of all patients with CRC develop peritoneal carcinomatosis during the natural course of their disease as a common sign of tumor progression or recurrence. In contrast to lymphatic and hematologic spread of metastases, intraperitoneal carcinomatosis develops by direct transcolonic tumor spread or tumor cell seeding during surgical resection of the primary tumor [5-8]. Tumor cell distribution within the abdominal cavity results in avascular tumor nodules that often cannot be efficiently addressed by systemic chemotherapy [9]. Thus, peritoneal carcinomatosis is mostly associated with poor prognosis. In the prospective European multicenter EVOCAPE 1 study, a median survival of 5.2 months was reported out of the 118 patients with peritoneal carcinomatosis arising from CRC during the natural course of disease [10]. Another retrospective analysis of 3,000 patients with peritoneal colon cancer dissemination reported a comparable median survival of 7 months [11].

### First-line treatment of advanced colorectal cancer

Systemic chemotherapy for metastatic colorectal cancer (mCRC) is mainly based on 5-FU with folinic acid (FA), preferably given as 24–48 h infusion, or oral prodrugs (e.g. capecitabine) in combination with either oxaliplatin or irinotecan [12]. Several studies with different chemo doublets could show median overall and progression-free survival ranging from 15 to 23 and 7 to 14 months, respectively, in patients with metastatic colorectal cancer (Table 1). Recently, Falcone et al. has shown a triple chemotherapy regimen combining 5-FU/FA, oxaliplatin and irinotecan (FOLFOXIRI) to be superior to FOLFIRI as first-line therapy [13]. In addition, triplets including targeted therapy such as antibodies against the vascular endothelial growth factor, VEGF (bevacizumab) or the epidermal growth factor receptor EGFR (cetuximab or panitumab) have been proven to be efficient in terms of prolonged overall and disease-free survival in first line mCRC treatment [14]. Thus, PFS reached up to 12 months and OS ranged from 17 to 30 months (Tables 1 and 2). Nevertheless, the efficacy of the different triplet regimens may depend on tumor biology-related factors (e.g. histology, dissemination pattern, KRAS or BRAF mutation, anticipated chemosensitivity and growth dynamics). However, triplets are recommended by the recently published ESMO Consensus Guidelines for first-line treatment or induction therapy for most of patients with advanced colorectal cancer [12].

**Table 1:** Selected RCTs for systemic chemotherapy of advanced colorectal cancer

**Author, year**	**Pat. [n]**	**Treatment regimen**	**Median PFS [months]**	**Median OS [months]**
DeGramont, 2000 [15]	210	FOLFOX4	9.0	16.2
Giacchetti, 2000 [16]	100	FOLFOX	8.7	19.4
Douillard, 2000 [17]	199	FOLFIRI	6.7	17.4
Saltz, 2000 [18]	226	IFL	7.0	14.8
Tournigand, 2003 [19]	109	FOLFIRI/FOLFOX6	14.2	21.5
Hurwitz, 2004 [20]	402	IFL/bevacizumab	10.6	20.3
Goldberg, 2006 [21]	154	FOLFOX4	9.7	19.0
Falcone, 2007 [13]	122	FOLFOXIRI	9.8	22.6
Fuchs, 2008 [22,23]	57	FOLFIRI/bevacizumab	11.2	28.0
Saltz, 2008 [24]	699	FOLFOX4/bevacizumab	9.4	21.3
Hecht, 2009 [25]	823	FOLFOX/FOLFIRI/bev	11.4	24.5
		+ panitumumab	10.0	19.4
Tebbutt, 2010 [26]	157	capecitabine/bevacizumab	8.5	-
Stathopoulos, 2010 [27]	114	IRI/5-FU/FA/bevacizumab	-	22.0
	108	IRI/5-FU/FA	-	25.0
Douillard, 2010 [28]	1183	FOLFOX4/panitumumab	9.6	23.9

**Table 2:** Cetuximab for advanced colorectal cancer

**Author, year**	**Pat. [n]**	**Treatment regimen**	**Median PFS [months]**		**Median OS [months]**	
			+ **cet**	- **cet**	+ **cet**	- **cet**
Tabernero, 2007 [29]	43	FOLFOX/cetuximab	12.3	-	30.5	-
Borner, 2008 [30]	74	CAPOX/cetuximab	7.2	5.8	20.5	16.5
Arnold, 2008 [31]	49	FUFOX/cetuximab	8.1	-	28.2	-
Cartwright, 2008 [32]	70	CAPIRI/cetuximab	8.1	-	20.5	-
Van Cutsem, 2009 [33]	348*	FOLFIRI/cetuximab	9.9*	8.7*	24.9*	21*
Bokemeyer, 2009 [34]	134*	FOLFOX/cetuximab	7.7*	7.2*	-	-
Tol, 2009 [35]	755	CAPOX/bev/cet	9.4	10.7	19.4	20.3
Maughan, 2011 [36]	729*	FOLFOX/CAPOX/cet	8.6*	8.6*	17.0*	17.9*
Tveit, 2012 [37]	566	FLOX/cetuximab	8.3	7.9	19.7	20.4

*wild-type KRAS.

### EGFR-targeted therapy for advanced colorectal cancer

The addition of targeted anticancer drugs against the epidermal growth factor receptor (EGFR), the monoclonal antibodies cetuximab and panitumumab, has further improved patient outcome in advanced stage colorectal cancer (Tables 2). Two prospective trials showed a survival benefit by adding cetuximab to best supportive care in patients with chemotherapy-refractory mCRC leading to a median OS of 6.4 and 6.1 months, respectively [38,39]. The BOND trial assigned patients with disease progression within three months after irinotecan-based chemotherapy to receive cetuximab with or without irinotecan. The median OS was 8.6 and 6.9 months, the time to progression 4.1 and 1.5 months, respectively [40]. In the randomized phase III CRYSTAL study investigating first-line treatment of mCRC the median PFS in the wild-type KRAS subgroup was 9.9 months in the FOLFIRI/cetuximab arm versus 8.7 months in the FOLFIRI arm. Median OS was 24.9 and 21 months, respectively. In patients with mutant KRAS status (n = 192) median PFS was reduced after additional treatment with cetuximab (7.6 vs. 8.1 months) [33,41]. Similar observations are reported by Bokemeyer et al. after subgroup analysis of the prospective randomized OPUS study. The median progression-free survival rate was 7.2 months in both treatment arms with a 0.5 months benefit for additional treatment with cetuximab in the wild-type KRAS subgroup [34]. The results have been confirmed by a recently published pooled analysis of the CRYSTAL and OPUS trials [42].

Moreover, these observations are supported by the PRIME study that showed a significant improvement of PFS of untreated patients with wild-type KRAS mCRC by adding the EGFR antibody panitumumab to FOLFOX-4. Median PFS was 9.6 months in the panitumumab group vs. 8.0 months in the control group. There was also a non-significant benefit in overall survival (23.9 vs. 19.7 months) [28]. Another prospective randomized phase III study showed an increased PFS after adding panitumumab to FOLFIRI in second-line treatment of patients with mCRC (5.9 vs. 3.9 months) [43].

In contrast, the MRC COIN trial investigating the addition of cetuximab to an oxaliplatin-based chemotherapy for first-line treatment of patients with advanced CRC could not reproduce these findings. Although the response rate increased from 57% to 64% by adding cetuximab there was no significant benefit in median OS (17.9 in the control group vs. 17.0 months in the cetuximab group) as well as PFS (8.6 vs. 8.6 months). Nevertheless, in the subgroup analysis the lack of benefit was only reported for oxaliplatin and fluoropyrimidine combinations plus cetuximab in contrast to combinations with infusional 5-FU [36]. In the recently published NORDIC-VII trial no benefit could be shown for the addition of cetuximab to an oxaliplatin-based combination with bolus 5-FU only (FLOX). In the ITT analysis the median progression-free survival was 7.9 months in the control group vs. 8.3 months in the cetuximab group, respectively [37].

### Systemic treatment of colorectal PM

Due to the fact that peritoneal metastases (PM) differ from other metastatic sites regarding clinical course and prognosis, Franko et al. published a pooled subgroup analysis of 364 patients with mCRC from the two North Central Cancer Treatment Group phase III trials N9741 and N9841. The patients were treated with oxaliplatin- or irinotecan-based systemic chemotherapy. The 5-year OS was 4.1% in patients with (additional) peritoneal metastases (PM) vs. 6% in patients without PM, showing a 30% relative reduction of OS in case of peritoneal carcinomatosis. Systemic chemotherapy with FOLFOX was superior to irinotecan-based treatment regimens, irrespective of the carcinomatosis status [44]. Klaver et al. analyzed the survival of the subgroup of patients with PM at the time of enrolment in the prospective clinical trials CAIRO and CAIRO2. The median OS significantly decreased in the PM group compared to patients without peritoneal tumor spread (CAIRO: 10.4 vs. 17.3 months, CAIRO2: 15.2 vs. 20.7 months) [45]. In 63 patients with colorectal PM selected from the French database that received several regimens of modern systemic chemotherapy the median OS was 23.9 months [46]. An Asian prospective single-arm phase II study investigating FOLFOX-4 in patients with peritoneal metastases from CRC reported a median time to progression of 4.4 months and a median overall survival of 21.5 months [47].

### Cytoreductive surgery and HIPEC

The combined treatment concept of cytoreductive surgery (CRS) and hyperthermic intraperitoneal chemotherapy (HIPEC) was introduced by Sugarbaker et al. in the early 1990’s and consists of complete macroscopic cytoreduction of all visible tumor nodules followed by local intraabdominal chemoperfusion at 41-42°C [48,49]. The aim of HIPEC in patients with peritoneal carcinomatosis is to circumvent the peritoneal barrier and to obtain higher local concentration of the cytostatic agents [50-52]. However, until today the intraperitoneal or bidirectional chemotherapeutic regimen is not standardized [53-56]. The addition of hyperthermia may potentiate the effect of the cytostatic agents by thermic cytotoxicity and induction of apoptosis. Moreover, heating can improve tissue penetration of the cytostatic agents [48,57,58].

Numerous retrospective analyses reported feasibility, safety and efficacy of the combined treatment concept of CRS and HIPEC in patients with peritoneal carcinomatosis arising from CRC (Table 3). However, data from prospective trials are still limited. Verwaal et al. reported a prospective randomized phase III trial analyzing CRS and HIPEC with MMC plus adjuvant chemotherapy with 5-FU/folinic acid compared to systemic chemotherapy with 5-FU/folinic acid and palliative surgery, if possible. After a median follow-up of 21.6 months, the experimental treatment arm showed a median overall survival of 22.3 months compared to 12.6 months in the standard arm. In the subgroup of patients with complete macroscopic cytoreduction (CC-0/1) median survival was 42.9 months. Median progression-free survival was 12.6 and 7.7 months, respectively [59,60]. Another randomized controlled trial was launched by a French group. This study published by Elias et al. was designed to compare CRS with early postoperative intraperitoneal chemotherapy (EPIC) to CRS alone. After premature termination due to recruitment difficulties a 2-year survival rate of 60% was reported in 35 patients with complete macroscopic cytoreduction [61]. In the comparative study published by Mahteme et al. the median survival in the HIPEC group was 32 months vs. 14 months in the control group. 5-year survival rates were 28% and 5% respectively [62]. A multicenter registry study of 506 patients treated with CRS and HIPEC for peritoneal carcinomatosis arising from colorectal cancer reported median overall survival of 19.2 months. In patients with complete macroscopic cytoreduction (CC-0/1) the median survival was 32.4 months [63]. In numerous observational studies the overall median survival ranged from 15 to 32 months and from 28 to 60 months after complete macroscopic cytoreduction (CC-0/1), respectively [64]. Elias et al. compared 48 patients from the French Multicenter Database with peritoneal carcinomatosis arising from CRC who received palliative systemic chemotherapy to 48 patients who underwent additional CRS and bidirectional oxaliplatin-based HIPEC. The chemotherapeutic regimen and the duration of systemic chemotherapy were comparable in both groups. The median survival was 23.9 months in the control group vs. 62.7 months in the HIPEC group, and the 5-year survival rate was 13% and 51%, respectively [46]. Comparable results were obtained in a recently published Belgian prospective multicenter phase II study in 48 consecutive patients with CRC and peritoneal carcinomatosis after CRS and oxaliplatin-based HIPEC. Hompes et al. reported a median time until recurrence of 19.8 months, and a 2-year overall survival rate of 88.7% [65]. The differences in median survival of the control group between these analyses and the Dutch Trial may be explained by patient selection and the introduction of more efficient combined chemotherapeutic regimens with or without targeted drugs in the standard treatment of advanced stage CRC.

**Table 3:** CRS and HIPEC

**Author, year**	**Pat. [n]**	**Cytostatic agents (HIPEC)**	**Median OS [mths]**	**Median PFS [mths]**	**OS [%]**	**Survival CC**-**0**/**1 [%]**
Pilati, 2003 [66]	34	MMC/DDP	18	-	31 (2-y)	-
Glehen, 2004 [67]	53	MMC	13	-	32 (2-y)	54 (2-y)
Glehen, 2004 [63]	506	MMC/LOHP	19	-	39 (3-y)	47 (3-y)
Shen, 2004 [68]	77	MMC	16	-	25 (3-y)	44 (3-y)
Verwaal, 03/08 [59,60]	105	MMC	22	12.6	28 (3-y)	45 (5-y)
Quenet, ASCO 08 [69]	37	LOHP/IRI	37	13	-	58 (3-y)
Elias, 2009 [46]	48	LOHP	63	-	-	51 (5-y)
Hompes, 2012 [65]	48	LOHP	-	19.8	-	89 (2y)

## Methods/design

### Study design

The COMBATAC study is a prospective, multicenter, open-label, single-arm, single-stage phase II study. The investigator initiated trial (IIT) is conducted by the Department of Surgery of the University Medical Center Regensburg in collaboration with the Center for Clinical Studies Regensburg, the Coordination Centre for Clinical Trials Duesseldorf and the participating national peritoneal carcinomatosis centers.

The study protocol is supported by the CRC Study Group of the Arbeitsgemeinschaft Internistische Onkologie (AIO) and the Chirurgische Arbeitsgemeinschaft Onkologie (CAO-V) of the German Society of General and Visceral Surgery (DGAV).

### Study objectives and endpoints

The primary objective of the COMBATAC study in patients with peritoneal carcinomatosis arising from wild-type KRAS colorectal and appendiceal cancer is to estimate the progression-free survival (PFS). Based on this estimation, it will be determined whether the multimodality treatment with pre- and postoperative systemic chemotherapy plus cetuximab, cytoreductive surgery (CRS) and bidirectional hyperthermic intraoperative chemotherapy (HIPEC) shows sufficient evidence of efficacy for further investigation.

PFS is defined as the time interval between the first day of preoperative treatment and the date of progression or death, whichever occurs first. Patients who are alive and progression-free at the time of analysis will be censored for PFS at the time of their last contact.

Secondary endpoints include overall survival, morbidity and toxicity related to the locoregional approach, feasibility of the combined treatment concept, quality of life and pathohistological regression.

### Study population

The study population of the COMBATAC study consists of patients with synchronous or metachronous peritoneal carcinomatosis arising from histologically proven wild-type KRAS colorectal or appendiceal cancer. The extent of peritoneal tumor spread (Peritoneal cancer Index, PCI) as assessed by diagnostics such as computed tomography and laparoscopy prior to patient enrolment should allow complete macroscopic cytoreduction (CC-0/1) at the time of surgery. Moreover, patients to be included in the study must meet the following inclusion criteria: treatment-free interval of at least 6 months after the completion of 3prior systemic chemotherapy, age over 18 and below 71 years, good general health status (Karnofsky index more than 70%, ECOG 0–2), absence of hematogenous metastases (lung, bone, brain, >3 peripheral resectable liver metastases), absence of contraindication for systemic chemotherapy and/or extended surgery, estimated life expectancy more than 6 months, absence of any psychological, familial, sociological or geographical condition potentially hampering compliance with the study protocol and follow-up schedule, written informed consent, creatinine clearance > 50 ml/min, serum creatinine ≤ 1.5 × ULN, serum bilirubin ≤ 1.5 × ULN, ASAT and ALAT ≤ 2.5 × ULN, platelet count > 100,000/ml, haemoglobin > 9 g/dl, neutrophil granulocytes ≥ 1,500/ml, International Normalized Ration (INR) ≤ 2, absence of peripheral neuropathy > grade 1 (CTCAE version 4.0), no pregnancy or breast feeding and adequate contraception in fertile patients. Patients with incomplete cytoreduction (≥CC-2), tumor debulking or palliative surgery, hematogenous metastasis excluding less than three resectable liver metastases and/or prior chemotherapy < 6 months before evaluation of study inclusion or therapy with EGFR receptor antibody for metastatic disease are excluded from the present study. Further exclusion criteria are KRAS mutation, known allergy to murine or chimeric monoclonal antibodies, concurrent chronic systemic immune therapy, chemotherapy, or hormone therapy not indicated in the study protocol, histology of signet ring carcinoma (>20% of tumor cells), other malignancy than disease under study or second cancer < 5 years after R0 resection, impaired liver, renal or hematologic function as mentioned above, heart failure NYHA ≥ 2 or significant coronary artery disease (CAD), alcohol and/or drug abuse, inclusion in other clinical trials interfering with the study protocol. Patients can only be included once in the COMBATAC study.

### Treatment schedule

**Figure 1:**
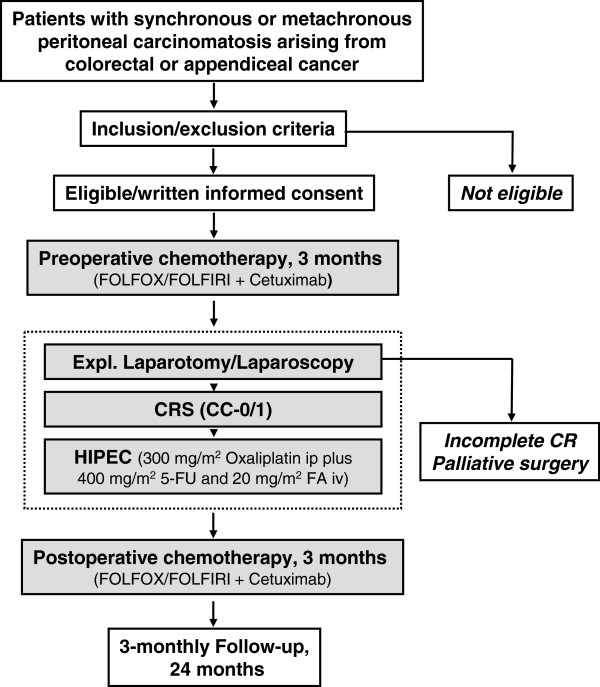
Flowchart of the COMBATAC study.

The interdisciplinary combined treatment regimen consists of pre- and postoperative systemic chemotherapy with FOLFOX or FOLFIRI plus the EGFR antagonist cetuximab, cytoreductive surgery (CRS) with complete macroscopic cytoreduction (CC-0/1) followed by bidirectional hyperthermic intraperitoneal chemotherapy (HIPEC). The treatment schedule is shown in Figure 1.

Systemic chemotherapy will consist of standard-of-care chemotherapy. Preoperative intravenous chemotherapy will be applied for 3 months, and therapy will be completed by postoperative systemic chemotherapy for further 3 months starting 4–6 weeks after surgery. Cetuximab is given intravenously once weekly for max. 12 weeks. The initial dose is 400 mg/m^2^ body surface area followed by a weekly dose of 250 mg/m^2^. Standard of care premedication will be administered as needed to patients receiving intravenous chemotherapy, including dexamethasone, acid suppressors, anti-emetics, analgetics and antipyretics. Systemic chemotherapy will be administered by the patients’ medical oncologist or the department of oncology of the enrolling peritoneal carcinomatosis center. All decisions regarding the management of (serious) adverse events related to systemic chemotherapy, such as dose reduction, interruption of systemic treatment or change of treatment regimen are at the discretion of the treating medical oncologist and are allowed within the study protocol, if documented.

Preoperative systemic chemotherapy is followed by cytoreductive surgery and HIPEC. The intent of cytoreductive surgery is to obtain complete macroscopic cytoreduction (CC-0/1) as a precondition for the application of HIPEC. The residual disease is classified intraoperatively using the completeness of cytoreduction (CC) score. CC-0 indicates no visible residual tumor and CC-1 residual tumor nodules ≤ 2.5 mm. CC-2 and CC-3 indicate residual tumor nodules between 2.5 mm and 2.5 cm and > 2.5 cm, respectively [70]. The initial extent of peritoneal tumor manifestation is determined intraoperatively using the Peritoneal Cancer Index (PCI, Washington Cancer Center), a combined numerical score of lesion size (LS-0 to LS-3) and tumor localization (region 0–12) [70,71]. During surgery patients are placed in modified lithotomy position. Surgery may include parietal and visceral peritonectomy, greater omentectomy, splenectomy, cholecystectomy, resection of liver capsule, small bowel resection, colonic and rectal resection, (subtotal) gastrectomy, lesser omentectomy, pancreatic resection, hysterectomy, ovariectomy and urine bladder resection. In patients with infiltration of the umbilicus, omphalectomy is necessary. Further operating procedures and resections may be necessary due to the intraoperative findings. Gastrointestinal reconstructions are performed following the individual center’s standard operating procedures (SOPs). The following minimal requirements are prerequisites for CRS: complete greater omentectomy, complete adhesiolysis of the small intestine, complete mobilization of the liver to assess the right diaphragmatic space, assessment of the left diaphragmatic space requiring splenectomy in the majority of cases, assessment of the left and right paracolic spaces, assessment of the pelvis, often requiring anterior rectal resection.

Bidirectional oxaliplatin-based hyperthermic intraperitoneal chemoperfusion (HIPEC) will only be applied intraoperatively in case of complete macroscopic cytoreduction (CC-0/1). HIPEC may be performed in an open or closed abdomen technique according to the peritoneal carcinomatosis center’s SOPs. After CRS four intraabdominal drains and two temperature probes are placed for continuous abdominal perfusion using a roller pump system with heat exchanger as described before [72]. When Douglas pouch temperature reaches 40°C oxaliplatin at a concentration of 300 mg/m^2^ body surface area is added and perfusion will be continued for further 30 minutes. The treatment is combined with synchronous IV administration of 400 mg/m^2^ fluorouracil and 20 mg/m^2^ folinic acid considering toxicity and safety instructions. After completion of the intraperitoneal perfusion cycle, the perfusion volume is evacuated from the abdominal cavity, all drains remain in situ and the patient is transferred to postoperative care.

### Assessments and follow-up

During the screening period patients will be assessed for eligibility to be included in the COMBATAC study. Inclusion and exclusion criteria are assessed by the investigator and initial diagnostics will be completed as necessary prior to patient enrolment. During pre- and postoperative systemic chemotherapy clinical examination and laboratory testing will be performed within 7 days of each chemotherapy cycle. After completion of preoperative treatment and after completion of the postoperative chemotherapy (end of treatment period), a further staging computed tomography will be performed. Moreover, quality of life is assessed and tumor markers (CEA, CA19-9) are determined. The same items will be recorded within three weeks after surgery. Intraoperative data consisting of PCI, surgical procedures, number of anastomoses, operating time, blood loss and course of HIPEC procedure and additional postoperative such as stay on ICU and hospital stay will be documented. The follow-up time starts 30 days after the last day of drug administration during postoperative treatment with the ‘end-of-treatment’ visit. The follow-up time takes 24 months with three-monthly follow-up visits consisting of physical examination, laboratory testing including tumor markers and protocol CT scans. Quality of life will be assessed yearly during follow-up.

Radiological disease progression will be assessed according to the revised RECIST criteria version 1.1 [73]. As mentioned above, computed tomography of the chest, abdomen and pelvis with oral, rectal and intravenous contrast will be performed prior to treatment start, within 3 weeks after cytoreductive surgery (CRS) and HIPEC and 30 days after the last systemic drug administration. Response to treatment is defined by the following four categories. (1) complete response (CR), (2) partial response (PR, 30% decrease in sum of baseline), (3) stable disease (SD) and (4) progressive disease (PD, new lesions or 20% increase in sum from nadir). Determination of disease progression in the absence of lymphatic or hematogenous disease recurrence will be based on clinical signs or symptoms (e.g. malignant ascites, ureteral stenosis or bowel obstruction), radiological diagnosis (CT ± PET) and/or surgical evidence of progression during laparoscopy or laparotomy. In addition, CEA and CA19-9 will be routinely measured as mentioned above. An at least three fold increase in serum CEA or CA19-9 levels will be defined as progression.

Morbidity and toxicity will be assessed as the number of medical and surgical complications occurring during the treatment period. The severity of complications (Grade I-V) will be assessed and adverse events will be categorized using the CTCAE version 4.0 [74].

Quality of life will be assessed using the EORTC QLQ-C30 questionnaire. Functional and symptom scores will be calculated according to the standard scoring procedures [75]. Comparisons will be drawn with the score means of the reference population [76]. A second round of analyses will be performed in order to identify the proportion of patients at any assessment point with pronounced deficits in QoL as defined by score points < 50 on a 0 = very bad to 100 = very good scale [77].

The pathohistological regression after systemic chemotherapy is assessed and graded using the classification published by Dworak et al [78]. This classification system was originally generated to evaluate regression of rectal cancer after neoadjuvant radiotherapy and consists of different types of necrosis and fibrosis with specific changes of vascular and cellular morphology.

### Statistical considerations

The sample size was calculated using the primary endpoint, i.e. progression-free survival (PFS). Based on the literature, a median PFS of 10 months or less was considered to be of no further interest (treatment not promising). Alpha (one-sided) was set to 10% and Beta was set to 20% (acceptable error rates for phase II trials [79]). Assuming exponentially distributed progression times and a target median PFS of 14 months (treatment promising), at least 39 events (progressions or deaths) have to be observed. Equivalently, if the true median PFS is 14 months, 39 events will be sufficient to rule out a median PFS of 10 months, based on the one-sided 90% confidence interval (CI). The normal approximation used in the calculations is given by equation 3.2.7 of Lawless [80]. Assuming an accrual period of 12 months and a follow-up of at least 18 months from the last patient recruited, a minimum number of 51 patients will be required. With a lost-to-follow-up rate of maximal 15%, a total of 60 patients have to be included in the study.

The final analysis with respect to PFS will be done after 39 observed events. PFS distribution and median PFS time with the corresponding one-sided 90% CI will be estimated by means of the Kaplan-Meier method. The treatment will be considered worth further investigation if the lower bound of the CI is greater than 10 months. The primary analysis will be based on the intention-to-treat (ITT) analysis set that consists of all patients who entered the study.

A detailed description of statistical analysis methods will be given in the Statistical Analysis Plan which will be finalized prior to database lock.

### Data collection and quality assurance

Patient data are collected in an electronic case report form (eCRF) at the data centre of the Center for Clinical Studies Regensburg in collaboration with the Coordination Centre for Clinical Trials Duesseldorf. Consistency checks will be performed on newly entered forms and queries issued in case of inconsistencies. Archiving of trial documents and trial data is performed according to the internal SOPs of the Center for Clinical Studies Regensburg. The originals of all essential trial documents are filed in the Trial Master File (TMF) and archived for at least 15 years. The site-specific documents in the Investigator Site File (ISF) will be archived at the site for at least 15 years. On-site monitoring will be performed by an external CRO (multi-service-monitoring, Regensburg, Germany) adapted according to the site accrual.

### Ethical and legal aspects

The protocol will be conducted according to the guidelines of Good Clinical Practice (GCP) and the ethical principles described in the Declaration of Helsinki. The study protocol was approved by the leading ethic committee (Ethikkommission an der Universitaet Regensburg) and the associated ethics committees, and was also subject to authorization by the national competent authority (BfArM) as mandatory by federal law. The study was assigned the EudraCT number 2009-014040-11 and is registered at ClinicalTrials.gov (NCT01540344).

## Discussion

The COMBATAC study is designed to evaluate the feasibility and efficacy of CRS and bidirectional oxaliplatin-based HIPEC as an additional treatment option for selected patients within an interdisciplinary combined treatment concept consisting of standard-of care pre- and postoperative systemic chemotherapy.

It is beyond question that systemic chemotherapy is the standard of care in patients with advanced stage CRC and peritoneal carcinomatosis. Although the oncological outcome of patients with advanced stage CRC and also the subgroup of patients with peritoneal carcinomatosis has improved since the introduction of combined chemotherapeutic regimens and new drugs, results of systemic therapy for patients with peritoneal carcinomatosis are still unsatisfactory [44]. Thus, additional treatment options should be evaluated. The existing data show that CRS and HIPEC may improve long-term survival of selected patients with peritoneal carcinomatosis of colonic origin [59,60]. Moreover, hyperthermic peritoneal perfusion with oxaliplatin in combination with synchronous intravenous application of 5-FU/folinic acid seems to improve the efficacy of HIPEC in comparison to a mitomycin C-based intraperitoneal treatment regimen, and may additionally contribute to a better local disease control [46,65]. Perioperative morbidity and mortality seems not to be impaired by the intensified oxaliplatin-based HIPEC regimen [81]. Nevertheless, the time of surgery including HIPEC, the perioperative treatment and the sequence of the therapeutic interventions is still a matter of debate. The intensified systemic treatment strategy with preoperative chemotherapy may lead to increased rates of complete macrosopic cytoreduction and together with the postoperative treatment to better control of distant metastasis and tumor recurrence. However, there is no prospective study available evaluating the clinical and oncological outcome after standard-of-care chemotherapy including targeted anticancer therapy in combination with CRS and HIPEC. Thus, the COMBATAC study is expected to give further information about the efficacy of this promising therapeutic option as an inherent part of a multidisciplinary treatment concept.

## Conclusions

To our knowledge the COMBATAC study is the first prospective clinical trial investigating the feasibility and efficacy of CRS and bidirectional oxaliplatin-based HIPEC within an interdisciplinary treatment regimen with pre- and postoperative systemic chemotherapy including cetuximab.

## Abbreviations

5-FU: Fluorouracil; BfArM: Bundesinstitut fuer Arzneimittel und Medizinprodukte; CA19-9: Carbohydrate antigen 19–9; CAPIRI: Capecitabine + irinotecan; CAPOX: Capecitabine + oxaliplatin; CEA: Carcinoembryonic antigen; COMBATAC: COMBined Anticancer Treatment of Advanced Colorectal cancer; CRC: Colorectal cancer; CRO: Contract research organization; CRS: Cytoreductive surgery; CT: Computed tomography; CTCAE: Common Terminology Criteria for Adverse Events; DDP: Cisplatin; EGFR: Epithelial growth factor receptor; EORTC: European Organisation for Research and Treatment of Cancer; EudraCT: European Clinical Trial Database; FA: Folinic acid; FOLFIRI: Folinic acid + fluorouracil + irinotecan; FOLFOX: Folinic acid + fluorouracil + oxaliplatin; HIPEC: Hyperthermic intraperitoneal chemotherapy; IFL: Irinotecan + fluorouracil + leucovorin; IIT: Investigator initiated trial; IRI: Irinotecan; ITT: Intention-to-treat; KRAS: Kirsten rat sarcoma viral oncogene homolog; LOHP: Oxaliplatin; mCRC: Metastatic colorectal cancer; MMC: Mitomycin C; MRI: Magnetic resonance imaging; OS: Overall survival; PCI: Peritoneal Cancer Index; PET: Positron emission tomography; PFS: Progression-free survival; PM: Peritoneal metastases; PP: Per protocol; QoL: Quality of life; RCT: Randomized controlled trial; SOPs: Standard operating procedures; ULN: Upper limit of normal.

## Competing interests

The COMBATAC study is financially supported by Merck KGaA, Darmstadt, Germany. GG, JR, SL, FZ and MK have nothing to declare. DA, FK, HJS and PP received honoraria from Merck KGaA.

## Authors’ contributions

GG drafted the manuscript and the study protocol. JR, DA, SAL, FK, FZ, MK, HJS and PP participated in writing the study protocol and revised the manuscript. PP is the principal and coordinating investigator of the COMBATAC trial. All authors read and approved the final manuscript.

## Pre-publication history

The pre-publication history for this paper can be accessed here:

http://www.biomedcentral.com/1471-2407/13/67/prepub
